# 784. Real-world effectiveness of the mRNA-12733-dose primary series against COVID-19 in an immunocompromised population Interim results from a prospective observational cohort study

**DOI:** 10.1093/ofid/ofac492.045

**Published:** 2022-12-15

**Authors:** Jennifer H Ku, Lina S Sy, Lei Qian, Bradley Ackerson, Yi Luo, Julia Tubert, Gina Lee, Ana Florea, Katia J Bruxvoort, Carla Talarico, Sijia Qiu, Yun Tian, Hung Fu Tseng

**Affiliations:** Kaiser Permanente Southern California, Pasadena, California; Kaiser Permanente Southern California, Pasadena, California; Kaiser Permanente Southern California, Pasadena, California; Kaiser Permanente Southern California, Pasadena, California; Kaiser Permanente Southern California, Pasadena, California; Kaiser Permanente Southern California, Pasadena, California; Kaiser Permanente Southern California, Pasadena, California; Kaiser Permanente Southern California, Pasadena, California; University of Alabama at Birmingham, Birmingham, Alabama; Moderna Inc., Cambridge, Massachusetts; Kaiser Permanente Southern California, Pasadena, California; Kaiser Permanente Southern California, Pasadena, California; Kaiser Permanente Southern California, Pasadena, California

## Abstract

**Background:**

While a 3-dose mRNA-1273 primary series is recommended for moderately or severely immunocompromised (IC) individuals in the U.S., some IC individuals do not complete the 3-dose series. We conducted a matched cohort study to evaluate the relative vaccine effectiveness (rVE) of the 3-dose mRNA-1273 primary series vs. 2 doses of mRNA-1273 in preventing SARS-CoV-2 infection and severe COVID-19 disease in IC individuals.

**Methods:**

IC individuals aged ≥18 years with ≥12 months of Kaiser Permanente Southern California membership who received 3 doses of mRNA-1273 ≥24 days apart were 1:1 matched with randomly selected IC 2-dose recipients on age, sex, race/ethnicity, and 2^nd^ dose date. Third doses were accrued from 08/12/2021 to 12/31/2021, with follow-up through 1/31/2022, spanning the delta and omicron periods. Outcomes were SARS-CoV-2 infection (positive molecular test or diagnosis code), COVID-19 hospitalization, and COVID-19 hospital death. Adjusted hazard ratios (aHR) with confidence intervals (CI) were estimated by Cox proportional hazards models. Adjusted rVE (%) was calculated as (1-aHR) x 100.

**Results:**

Our study included 21,942 3-dose and 21,942 2-dose mRNA-1273 IC recipients. Adjusted rVE of 3 doses compared to 2 doses of mRNA-1273 against SARS-CoV-2 infection, COVID-19 hospitalization, and COVID-19 hospital death were 55.0% (95% CI: 50.8–58.9%), 83.0% (75.4–88.3%), and 87.1% (30.6–97.6%), respectively (Table 1). Adjusted rVE against SARS-CoV-2 infection ranged from 43.0% to 59.1% across subgroups of age, sex, race/ethnicity, history of SARS-CoV-2 infection, pregnancy, and comorbidities (Table 2). Point estimates of the 3-dose rVE were higher against COVID-19 infection and hospitalization in the first 3 months, compared to 3–6 months after the 3^rd^ dose (Table 3).

**Conclusion:**

Three doses of mRNA-1273 provide additional protection against SARS-CoV-2 infection and severe outcomes for IC individuals, compared to 2 doses, highlighting the importance of completing 3-doses for IC populations. However, possible waning of protection against SARS-CoV-2 infection and severe outcomes after 3 months supports the ACIP recommendation of a booster dose at least 3 months after the 3^rd^ primary series dose for adequate protection of IC individuals.

**Disclosures:**

**Jennifer H. Ku, PhD MPH**, GSK: Grant/Research Support|Moderna: Grant/Research Support **Lina S. Sy, MPH**, Dynavax: Grant/Research Support|Glaxosmithkline: Grant/Research Support|Moderna: Grant/Research Support|Seqirus: Grant/Research Support **Lei Qian, PhD**, Dynavax: Grant/Research Support|Glaxosmithkline: Grant/Research Support|Moderna: Grant/Research Support **Bradley Ackerson, MD**, Dynavax: Grant/Research Support|Glaxosmithkline: Grant/Research Support|Moderna: Grant/Research Support|Pfizer: Grant/Research Support|Seqirus: Grant/Research Support **Yi Luo, PhD**, Glaxosmithkline: Grant/Research Support|Moderna: Grant/Research Support|Pfizer: Grant/Research Support|Seqirus: Grant/Research Support **Julia Tubert, MPH**, Moderna: Grant/Research Support|Pfizer: Grant/Research Support **Gina Lee, MPH**, Glaxosmithkline: Grant/Research Support|Moderna: Grant/Research Support **Ana Florea, PhD MPH**, Gilead: Grant/Research Support|GSK: Grant/Research Support|Moderna: Grant/Research Support|Pfizer: Grant/Research Support **Carla Talarico, PhD**, Moderna: Employee of and a shareholder in Moderna Inc. **Sijia Qiu, MS**, Dynavax: Grant/Research Support|Moderna: Grant/Research Support **Yun Tian, MS**, Glaxosmithkline: Grant/Research Support|Moderna: Grant/Research Support **Hung Fu Tseng, PhD MPH**, GSK: Grant/Research Support|Janssen: Advisor/Consultant|Moderna: Grant/Research Support|Pfizer: Advisor/Consultant|Seqirus: Grant/Research Support.

**Table 1.**
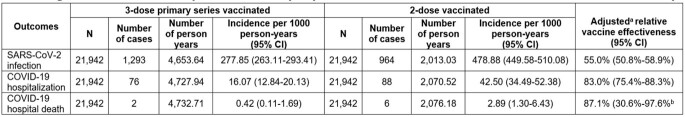
Incidence rate and relative vaccine effectiveness of the mRNA-1273 3-dose primary series in preventing SARS-CoV-2 infection, COVID-19 hospitalization, and COVID-19 hospital death among immunocompromised population, with 2-dose vaccinated comparison

a-Adjusted for covariates age, sex, race/ethnicity, index date (in months), time between second dose and index date, number of outpatient and virtual visits, preventive care, Charlson comorbidity score, and immunocompromising sub-conditions*.

b-Adjusted for covariates age, sex, index date (in months), time between second dose and index date, and immunocompromising sub-conditions* (except HIV/AIDS).

*Leukemia, lymphoma, congenital immunodeficiencies, asplenia/hyposplenia, HIV/AIDS, transplant (hematopoietic stem cell and solid organ), and/or receipt of immunosuppressive medications.

**Table 2.**
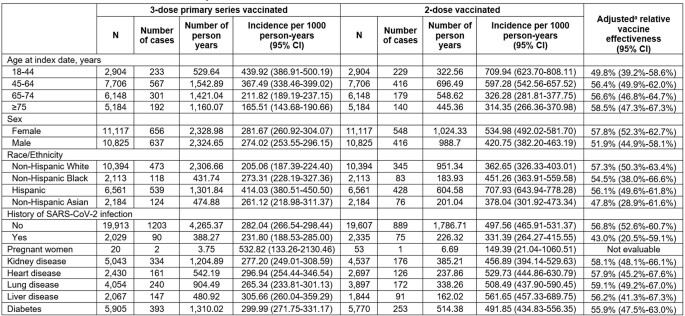
Incidence rate and relative vaccine effectiveness of the mRNA-1273 3-dose primary series in preventing SARS-CoV-2 infection by subgroups among immunocompromised population, with 2-dose vaccinated comparison

a-Adjusted for covariates age, sex, race/ethnicity, index date (in months), time between second dose and index date, number of outpatient and virtual visits, preventive care, Charlson comorbidity score, and immunocompromising sub-conditions (leukemia, lymphoma, congenital immunodeficiencies, asplenia/hyposplenia, HIV/AIDS, transplant [hematopoietic stem cell and solid organ], and/or receipt of immunosuppressive medications).

**Table 3.**
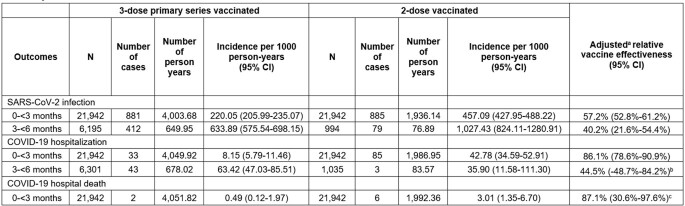
Incidence rate and relative vaccine effectiveness of the mRNA-1273 3-dose primary series in preventing SARS-CoV-2 infection, COVID-19 hospitalization, and COVID-19 hospital death by month after index date among immunocompromised population, with 2-dose vaccinated comparison

a-Adjusted for covariates age, sex, race/ethnicity, index date (in months), time between second dose and index date, number of outpatient and virtual visits, preventive care, Charlson comorbidity score, and immunocompromising sub-conditions*

b-Adjusted for covariates age, sex, index date (in months), preventive care, Charlson comorbidity score, and immunocompromising sub-conditions*

c-Adjusted for covariates age, sex, index date (in months), time between second dose and index date, and immunocompromising sub-conditions* (except HIV/AIDS)

Vaccine effectiveness (%) was calculated as (1 – hazard ratio) × 100 when the hazard ratio was less than or equal to 1; vaccine effectiveness (%) was calculated as ([1/hazard ratio] – 1) × 100 when the hazard ratio was greater than 1.

*Leukemia, lymphoma, congenital immunodeficiencies, asplenia/hyposplenia, HIV/AIDS, transplant (hematopoietic stem cell and solid organ), and/or receipt of immunosuppressive medications

